# Supplementary material for: Dose-response relationship between diarrhea quantity and mortality in critical care patients: A retrospective cohort study
Source: PLoS One. 2023 Feb 13;18(2):e0280806. doi: 10.1371/journal.pone.0280806 (PMC9925000; doi:10.1371/journal.pone.0280806)
Supplement: S1 Table — (DOCX) [file pone.0280806.s001.docx]

| Supplementary Table. The mortality and length of stay of the entire cohort | | | |
| --- | --- | --- | --- |
|  | Total  n=1579 | Non-diarrhea^†^  n=1245 | Diarrhea^‡^  n=334 |
| ICU mortality, n (%) | 103 (6.5) | 69 (5.5) | 34 (10) |
| In-hospital mortality, n (%) | 212 (13) | 126 (10) | 86 (26) |
| ICU LOS, median [IQR], day | 3.0 [2.0, 5.0] | 2.0 [2.0, 3.8] | 7.0 [4.0, 11.0] |
| Hospital LOS, median [IQR], day | 17 [9, 39] | 14 [8, 29] | 39 [19, 68] |
| †Patients who did not meet inclusion criteria, ‡Patients who met the inclusion criteria (including patients who met exclusion criteria). There was no missing outcome. IQR: Interquartile range, LOS: Length of stay, IQR: Interquartile range. | | | |
